# The relationship between low serum calcium level and intracerebral hemorrhage hematoma expansion: A protocol of systematic review and meta-analysis

**DOI:** 10.1097/MD.0000000000018844

**Published:** 2020-01-17

**Authors:** Jian Sun, Wanjun Liu, Rending Zhu, Yao Wu, Liqi Yang

**Affiliations:** The Second Affiliated Hospital of Anhui Medical University, Economic and Technological Development Zone of Hefei, Anhui Province, China.

**Keywords:** intracerebral hemorrhage, hematoma, hypocalcemia, serum calcium

## Abstract

**Background:**

To investigate the relationship between intracerebral hemorrhage hematoma expansion with low serum calcium level.

**Methods:**

We will search the following electronic bibliographic databases: MEDLINE, Embase, PubMed, The Cochrane Library, and Web of Science. All sources have to be searched from the earliest date until May 1, 2019. The quality of the included studies will assess by 2 evaluation members according to the Cochrane Collaboration network standard or the Newcastle–Ottawa Scale. The included studies will analysis by using RevMan 5.3 software.

**Results and conclusion:**

This will be the first systematic review and meta-analysis to evaluate the association of hematoma following intracerebral hemorrhage with hypocalcemia. The study will provide more reliable, evidence-based data for clinical decision making.

**Prospero registration number:**

CRD42019135956

## 1 Introduction

Spontaneous intracerebral hemorrhage (ICH) is one of the most severe types of stroke with high mortality and disability.^[1]^ Baseline hematoma volume and hematoma expansion (HE) are recognized prognostic factors for this disease.^[2,3]^ Studies have shown that low calcium levels are associated with higher baseline hematoma volume, higher HE risk, and worse outcome.^[4,5]^ However, there is a lack of evidence-based data on the relationship between low serum calcium level and ICH HE. The purpose of this study is to provide reference data for clinical decision making.

## 2 Methods

### 2.1 Standards

This protocol will be performed comply with the Preferred Reporting Items for Systematic Reviews and Meta-Analyses Protocols (PRISMA-P) guidelines.

### 2.2 Ethical issues

Ethical approval was not necessary because this was a systematic review and meta-analysis based on published data.

### 2.3 Registration

The protocol has been registered on PROSPERO with number: CRD42019135956.

### 2.4 Inclusion criteria

The inclusion criteria are spontaneous ICH that was detected by noncontrast computed tomography performed within 72 hours from the presumed symptom onset, and a total serum calcium measurement obtained on admission.

### 2.5 Exclusion criteria

The exclusion criteria are traumatic intracranial hemorrhage; intracranial tumor or vascular malformations presumed to be the cause of the hemorrhage; primary intraventricular hemorrhage; and hemorrhagic conversion of acute ischemic stroke.

### 2.6 Search strategy

We will search the following electronic bibliographic databases: MEDLINE, Embase, PubMed, The Cochrane Library, and Web of Science. All sources have to be searched from the earliest date until May 1, 2019. And we will only include articles published in English. The search will be performed using multiple combinations of the following keywords: serum calcium OR blood calcium OR hypocalcemia; AND hematoma volume OR extent of bleeding OR hematoma expansion; AND intracerebral hemorrhage OR cerebral hemorrhage AND association OR relationship.

### 2.7 Data analysis and statistical methods

All data will be subjected to meta-analysis using Review Manager software (version 5.3.3; Cochrane Collaboration). Statistical heterogeneity will assess by Chi-squared and *I*^2^ tests. If the *I*^2^ value was >50%, the data will be considered to be significantly heterogeneous. Continuous data will be represented by mean differences (MDs) and 95% confidence intervals (CIs), while dichotomous data will be represented by odd ratios (ORs) and 95% CIs. A *P*-value of <.05 will be considered statistically significant.

### 2.8 Quality assessment

Two researchers will independently evaluate the quality of the literature. Studies will be evaluated using the Cochrane risk of bias tool and Newcastle–Ottawa scale.

## 3 Discussion

Calcium, as the most abundant mineral element in the human body, maintains the stability of the blood–brain barrier in the nervous system and participates in regulating the apoptosis of nerve cells.^[6–8]^ Studies have shown that hematomas in patients with cerebral hemorrhage with hypocalcemia are larger.^[5]^ In addition, larger hematoma volume has a negative effect on the prognosis of ICH, while higher blood calcium concentration is associated with better prognosis of ICH patients.^[9]^ The potential mechanisms are as follows: Calcium may have a potential role in regulating blood pressure: epidemiologic studies have shown that people who consume less calcium have a higher risk of developing hypertension^[10]^; Calcium is involved in tissue-factor activation and platelet adhesion during coagulation: animal studies have shown that blood clotting time is significantly prolonged in calcium-deficient rats.^[11]^ However, the relationship between hypocalcemia and spontaneous ICH hematoma volume has not been fully confirmed. Based on the results of previous studies, this study will analyze the correlation between hypocalcemia and hematoma volume in patients with ICH, to explore the potential value of hypocalcemia in predicting prognosis.

## Author contributions

**Conceptualization:** Jian Sun, Wanjun Liu, Rending Zhu.

**Data curation:** Jian Sun, Wanjun Liu, Rending Zhu, Yao Wu.

**Formal analysis:** Jian Sun, Wanjun Liu, Yao Wu.

**Funding acquisition:** Jian Sun, Wanjun Liu, Liqi Yang.

**Methodology:** Jian Sun, Wanjun Liu.

**Supervision:** Liqi Yang.

**Writing – original draft:** Jian Sun, Wanjun Liu, Rending Zhu.

**Writing – review & editing:** Jian Sun, Wanjun Liu, Liqi Yang.
